# *QuickStats:* Rate of Emergency Department Visits*^,^^†^ for Substance Use Disorders^§^ Among Adults Aged ≥18 Years, by Age Group — National Hospital Ambulatory Medical Care Survey, United States, 2018–2019 and 2020–2021

**DOI:** 10.15585/mmwr.mm7239a6

**Published:** 2023-09-29

**Figure:**
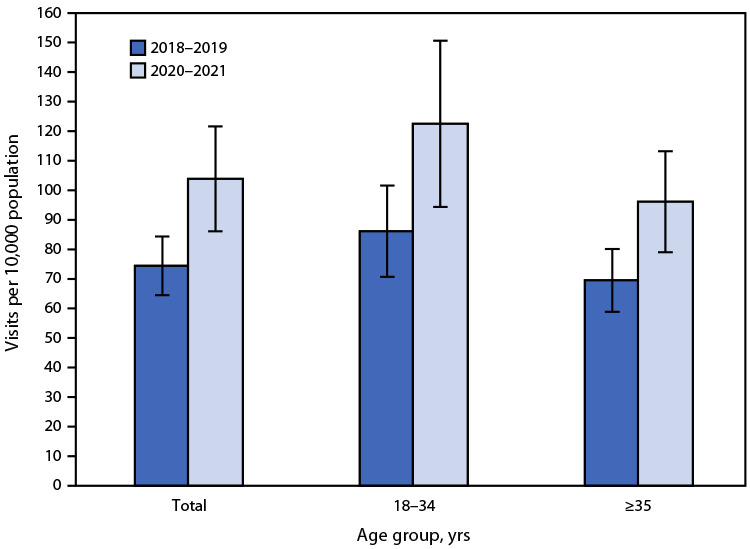
The rate of emergency department visits with a primary diagnosis of a substance use disorder among adults increased from 74.4 per 10,000 population during 2018–2019 to 103.8 during 2020–2021. Between these two periods, this rate increased 42% among patients aged 18–34 years (from 86.1 to 122.5) and 38% among patients aged ≥35 years (from 69.5 to 96.1). During both 2018–2019 and 2020–2021, adults aged 18–34 years were more likely to visit an emergency department for substance abuse, use, or dependence than were those aged ≥35 years.

**Source**: National Center for Health Statistics, National Hospital Ambulatory Medical Care Survey, 2018–2021. https://www.cdc.gov/nchs/ahcd/index.htm

For more information on this topic, CDC recommends the following link: https://www.cdc.gov/drugoverdose/featured-topics/substance-use-disorders/

